# A Lightweight and Versatile Prosthetic Hand for Daily Grasping

**DOI:** 10.3390/biomimetics11040257

**Published:** 2026-04-08

**Authors:** Shunping Zhao, Yuki Inoue, Zhenyu Chen, Yicong Lin, Junru Chen, E. Tonatiuh Jimenez-Borgonio, J. Carlos Sanchez-Garcia, Yinlai Jiang, Hiroshi Yokoi, Xiaobei Jing, Xu Yong

**Affiliations:** 1 Shenzhen Institutes of Advanced Technology, Chinese Academy of Sciences, Shenzhen 518055, China; sp.zhao@siat.ac.cn; 2 University of Chinese Academy of Sciences, Beijing 101408, China; 3 Sino-Japan Joint Laboratory for Human-Machine Interaction and Functional Rehabilitation, Shenzhen 518055, China; 4 Department of Mechanical Engineering and Intelligent Systems, The University of Electro-Communications, Tokyo 182-0021, Japan; y.inoue@hi.mce.uec.ac.jp (Y.I.); ejimenezb2200@alumno.ipn.mx (E.T.J.-B.); jiang@hi.mce.uec.ac.jp (Y.J.); yokoi@mce.uec.ac.jp (H.Y.); 5 College of Mechatronics and Control Engineering, Yuehai Campus, Shenzhen University, Shenzhen 518055, China; 2023111090@email.szu.edu.cn (Z.C.); 2023111100@email.szu.edu.cn (Y.L.); 2023111070@email.szu.edu.cn (J.C.); 6 Seccion de Estudios de Posgrado e Investigacion, Escuela Superior de Ingenieria Mecanica y Electrica Culhuacan, Instituto Politecnico Nacional, Mexico City 04440, Mexico; jcsanche@ipn.mx

**Keywords:** prosthetic hand, tendon-driven transmission, encoder-based sensorless termination

## Abstract

To meet daily grasping needs under lightweight, low-complexity wearable constraints, this study proposes an underactuated multi-finger prosthetic hand with transmission–control co-design to achieve predictable multi-joint synergies and stable grasps under limited actuation. The prototype uses six miniature motors to drive 14 joint degrees of freedom (DOFs): four fingers have active metacarpophalangeal actuation with tendon-driven underactuated proximal and distal interphalangeal joints, while the thumb provides two independently controlled DOFs for opposition expansion and posture adjustment. It supports five-finger power grasps, tripod pinches, and lateral pinches. To mitigate tendon slack and stroke inconsistency, active/passive tendon-length constraints are defined, and an equal-stroke configuration is obtained via chord-to-arc mapping. A layered STM32F767-based controller combines a reference rotation range limit (free motion) with encoder speed-decay detection (contact/near-stall) to realize per-finger termination and overdrive protection without force/tactile sensors. Experiments report a total mass of 176.6 g and a peak single-finger driving force of approximately 2.8 N. Following the Feix GRASP taxonomy (33 types), the hand reproduces 24 types (72.7%), covering power, intermediate and precision grasps, both thumb abduction/adduction postures, and palm–pad–side opposition/contact, with stable grasp formation across objects of varying geometries.

## 1. Introduction

Multi-finger prosthetic hands are important in upper limb rehabilitation and assistive robotics. For daily assistive use, the design objective is to retain representative grasping and manipulation functions within strict constraints on volume and physical parameters. In this context, biomimetic design is better reflected in the functional organization of grasp formation and opposition than in a direct increase in structural complexity. Recent advances in miniature actuators, embedded control systems, and rapid manufacturing technologies have enabled prosthetic hands with increasingly rich grasping functions, and both research prototypes and commercial devices can now realize a broad range of representative grasp patterns. However, from both engineering and deployable perspectives, high functional coverage often comes at the expense of an increased actuator count, system mass and mechanical/control complexity. Such designs not only result in increased manufacturing and maintenance costs but may also affect long-term wearing comfort and system reliability [1,2,3]. Therefore, to preserve necessary grasping capability under a tightly constrained wearable budget remains a critical challenge in prosthetic hand design.

Against this background, underactuated biomimetic prosthetic hands have become a promising design direction with obvious engineering benefits. By using tendons, differential mechanisms, or compliant elements, underactuated structures can be used to coordinate multiple joints with a single actuator, to achieve coupled multi-joint motions without a proportional increase in the number of actuators [4,5,6]. For daily grasping tasks, such designs exploit mechanically embodied passive adaptability, reaching grasp stability and success rates comparable to fully actuated hands while greatly reducing system weight and energy consumption [7]. Nevertheless, existing implementations of the technology are still polarized; some approaches focus on extreme actuation simplification to achieve compactness but have limited controllability over task-critical grasp postures. Others improve the completeness of functions but are based on complex differential mechanisms or additional actuators and lose the advantages of lightweightness and wearability inherent in underactuation [8]. These observations show that it is not enough to reduce the number of actuators because it is not possible to simultaneously meet the requirements of functional coverage and engineering feasibility.

These observations also suggest that the key issue in underactuated prosthetic hands is not merely whether actuation can be reduced, but how the limited actuation should be distributed to preserve functionally meaningful grasping. Existing studies have explored several representative routes. Actuation-oriented analyses have shown that the functionality of underactuated anthropomorphic hands depends strongly on how actuation is allocated across the fingers and thumb, because different allocation strategies lead to markedly different trade-offs between grasp diversity and functional usefulness [9]. Representative prototypes have therefore adopted distinct solutions, including lock-based tendon-pulley architectures that expand grasping capabilities through selectively constrained finger motion [10], mechanically programmable underactuated prosthetic grippers that combine tendon-driven fingers with reconfigurable thumb behavior [11], and synergy-inspired low-actuator hands that emphasize compactness together with simplified multimodal control [12]. Taken together, these studies confirm the engineering value of under-actuation, but they also indicate that lightweight implementation, representative grasp-type differentiation, and low-complexity control are often optimized only partially rather than under unified allocation logic. In this sense, an important unresolved issue in underactuated prosthetic hands is how limited actuation should be allocated across the hand so that coordinated grasp formation remains functionally meaningful while still satisfying lightweight and engineering constraints.

Under constrained actuation, single-finger architecture plays an important role in achieving coordinated multi-joint motion with adequate predictability and stability. In underactuated prosthetic fingers, a common engineering strategy is to preserve direct control over the dominant flexion component while using mechanical coupling to coordinate the remaining joints, so that synergistic multi-joint flexion can be achieved without a proportional increase in actuator count. Embedding explicit mechanical constraints to predefine inter-joint motion relationships allows such architectures to produce more stable and interpretable coordination patterns during flexion, thereby reducing the need for complex control algorithms. Compared with fully independently actuated designs, this method has clear advantages in compactness and mass reduction. Compared with highly coupled or single-input structures, it also preserves more interpretable and functionally separable postures, which not only benefits configuration reproduction and grasp envelopment, but also provides the structural basis for representative daily grasp formation under constrained actuation [13,14,15,16,17,18,19,20].

Such grasp formation relies on three closely related functional features. The first is coordinated closure of the four fingers, in which grasp formation is dominated by low-dimensional flexion patterns rather than fully independent joint regulation. The second is post-contact redistribution, where mechanical coupling and compliance support adaptive envelopment as contact conditions change. The third is thumb-side opposition adjustment, which determines opposition location and contact mode and thereby strongly influences the separation of representative grasp types. These features are directly reflected in the present combination of four-finger underactuated closure and a preserved two-DOF thumb configuration, in which thumb-side opposition adjustment remains the key geometric factor for differentiating representative grasp types under limited actuation.

Thumb-side DOF allocation has a decisive influence on grasp-type differentiation in a prosthetic hand. Opposition adjustment provided by the thumb is fundamental to the formation of stable pinch-like and precision grasp configurations with the four fingers [21]. Increasing thumb DOFs can broaden grasp-posture diversity, but it usually requires additional actuators or more complicated mechanisms, which conflicts with the goal of lightweight design [22,23]. Conversely, excessive simplification of thumb motion can severely reduce practical utility in common grasping situations [24]. Therefore, under constrained actuation, the engineering question is not simply whether more thumb mobility is beneficial, but which thumb-side adjustments exert the strongest influence on opposition geometry and the grasp plane and should thus be preferentially preserved [25,26,27,28].

At the control level, although underactuated mechanisms offer passive mechanical adaptability, reliable grasp execution still requires effective state estimation and termination mechanisms to avoid overdriving and structural damage. Conventional approaches often rely on force, tactile, or slip sensing to support fine closed-loop grasp control [29]. In lightweight wearable prosthetic hands however, multi-sensor configurations can substantially increase system complexity, wiring burden, and potential failure points [30,31]. Under these constraints, a more practical route is to use available motor-side feedback, such as encoder position/velocity or current-related signatures, to infer contact and near-stall conditions [32,33,34,35,36,37,38]. Although this route is not intended for fine force regulation, it enables reliable grasp termination without introducing additional sensors, thereby reducing implementation complexity while supporting overall system robustness.

Recent prosthetic-hand studies have expanded grasp capability through substantially different technical routes, but the underlying distinction lies not only in how many functions are achieved, but also in where mechanical and control complexity is allocated. Highly articulated platforms typically increase active degrees of freedom and rely on richer sensing and control loops to extend dexterity [39], whereas semi-autonomous systems reduce user burden through multimodal sensing and autonomous grasp execution [40]. Other studies simplify the hand more uniformly at the whole-hand level, reducing actuation and control burden while retaining only a limited number of distinct grasp patterns in exchange for lower structural and control complexity [41]. In contrast, the present study does not pursue any of these routes directly. Instead, it adopts a selective-underactuation strategy in which complexity is reduced on the four-finger side through tendon-coupled underactuated closure, while thumb-side geometric adjustment is preserved and paired with an encoder-based sensor-minimal execution framework. The purpose of this allocation is not to maximize overall dexterity, but to retain representative grasp-type differentiation under simultaneous constraints on wearable mass, actuator count, and sensing configuration. This interpretation is also consistent with prior observations that thumb orientation and actuation strongly affect the realization of representative grasps, especially tripod and lateral grasps [42], and that differentiated drive allocation may be more effective than uniform simplification when both precision and conformal grasp behaviors need to be retained [43].

To address the tightly constrained wearable budget in actuator count, mass, and sensing requirements and retain functionally meaningful grasp-type differentiation, we propose a lightweight prosthetic hand based on transmission–control co-design. The design allocates one actuator to each of the four fingers and two actuators to the thumb, so that underactuated envelopment and thumb-mediated opposition can be organized in a complementary manner. At the finger level, the MCP joint is actively driven, whereas the PIP and DIP joints are coupled through a tendon-based underactuated mechanism to realize predictable synergistic flexion under limited actuation. At the hand level, the thumb is assigned two independently controlled DOFs to preserve the grasp-type differentiation required for representative daily grasps such as power grasp, tripod pinch, and lateral pinch. Rather than pursuing full anthropomorphic replication or high-DOF actuation, this work focuses on a reproducible engineering route for balancing lightweight design, grasp-type coverage, and control simplicity. The resulting prototype is evaluated through posture-reproduction and multi-object grasping experiments to examine whether this coordinated structural and control strategy can preserve broad functional coverage under constrained hardware conditions.

To address this engineering question, the main contributions of this work are summarized as follows:

(1) A selective-underactuation design strategy for lightweight multi-finger prosthetic hands is proposed. Instead of maximizing either DOF count or actuation simplification alone, the design assigns active MCP actuation to the four fingers and preserves two active thumb DOFs, thereby explicitly targeting the balance between wearable lightweight constraints and grasp-type differentiation.

(2) An interpretable tendon-transmission organization is developed for predictable coupled finger flexion. By formulating active/passive tendon-length constraints and implementing an equal-stroke actuation configuration, the proposed mechanism improves the consistency and reproducibility of underactuated multi-joint motion.

(3) A low-complexity sensorless grasp-formation framework is established using encoder-based state monitoring. Without introducing additional force or tactile sensors, the controller integrates reference rotation constraints and speed-decay-based contact/near-stall inference to support grasp termination and overdrive protection under underactuated grasping conditions.

(4) A prototype-level validation framework is provided through posture-reproduction and representative grasping experiments. The results show that broad functional coverage can still be achieved under limited actuation and sensing, while also clarifying the practical boundary between lightweight implementation and grasp-function coverage. 

The rest of this paper is organized as follows. Section 2 describes the overall design of the hand, the tendon-driven underactuation and transmission mechanism, and the encoder feedback-based control and grasp termination strategy. Section 3 introduces key performance results and multi-object grasping experiments, where action library-level coverage is evaluated based on the GRASP taxonomy. Section 4 deals with the structure–control synergy and examines the benefits and challenges of the proposed design. Finally, Section 5 summarizes the main findings and conclusions of the study.

## 2. Materials and Methods

### 2.1. Overall Hand Design Overview

**Figure 1:**
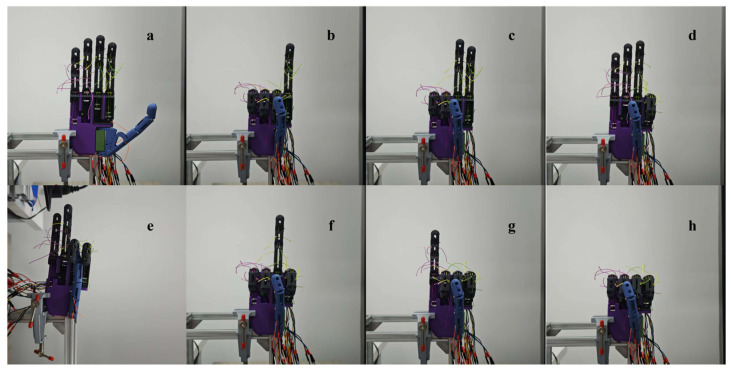
Physical prototype of the proposed prosthetic hand and representative multi-finger postures. (**a**) Four-finger extension; (**b**) index finger extension; (**c**) index and middle finger extension; (**d**) extension of the middle, ring, and little fingers; (**e**) ring–little finger extension; (**f**) middle finger extension; (**g**) little finger extension; (**h**) fist-like closure.

The physical prototype of the proposed prosthetic hand and representative multi-finger postures are shown in Figure 1. The hand is made of 5 digits and actuated by 6 miniature DC motors (N20, China): one motor is dedicated to actuating each of the index, middle, ring and little fingers, while the thumb is actuated by two independent actuators. This configuration gives a total of 14 mechanical DOFs, three joint DOFs for each of the four fingers and two DOFs for the thumb. Such a distribution allows independent flexion and extension of each digit, as well as coordinated multi-finger postures, which support representative grasp configurations including the five-finger power grasp and tripod pinch.

Once the actuation count and the DOF allocation are determined, the spatial arrangement of the actuators has a direct impact on the mass distribution and structural compact-ness of the hand. Accordingly, all drive motors are mounted at the dorsal (back-of-hand) side. This centralized arrangement close to the palm minimizes added mass on the distal segments of the fingers and maintains adequate space for structures of the intra-finger joints and tendon-routing paths. Furthermore, the dorsal placement ensures that the palmar side is unobstructed, there is no interference between actuation components and the object-contact region, and the tendon anchoring, replacement, and routine maintenance are simplified. The main geometric dimensions of the present prototype are summarized in Table 1, Table 2 and Table 3.

These dimensions define the geometric scale of the current adult-size prototype and provide the dimensional basis for the following structural description. In principle, the present tendon-driven underactuated mechanism is compatible with moderate geometric scaling; however, child-oriented or other size-specific versions would require dedicated redesign and validation rather than direct scaling of the current prototype, because the feasible scaling range is constrained by tendon-routing space, actuator arrangement, and transmission geometry.

For the single-finger design, the four fingers have a three-joint serial kinematic chain corresponding to the MCP, PIP, and DIP joints. Each MCP joint is directly actuated by a DC motor, and the PIP and DIP joints are passively coupled to the MCP joint via tendon transmission. During flexion of the finger, the PIP and DIP joints rotate synchronously with the MCP joint, causing coordinated multi-joint flexion. This coupled motion generates joint synergies that closely approximate the natural flexion pattern of the human finger, facilitating smooth and continuous fingertip motion during grasping. Detailed descriptions of the mechanical structure and coupling relationships of the finger are given in the following sections.

In contrast to the other four fingers, which are mainly involved in flexion and extension movements, the thumb is important for the establishment of opposition and the stabilization of object posture in multiple modes of grasp. To satisfy these functional requirements, the thumb has a dual-motor actuation scheme to allow posture adjustment for various grasps. Specifically, the motor that is closer to the base of the thumb is in charge of the rotational motion, which is in relation to the palm, to regulate spatial placement during the formation of the grasp, and the other motor that is further down the thumb is in charge of adduction and abduction, responsible for opposition to the other fingers. Through the coordination of these two independent DOFs, the thumb is able to make the required pose adjustments for representative grasp modes, such as the five-finger power grasp, tripod pinch, and lateral pinch.

DC motors are used for actuation applications because of their small size, low weight and high torque with integrated encoders to provide real-time feedback on the state of the motion. Motor output is transferred to the joints of the fingers by a tendon-driven mechanism. The tendons are passed along the inside of the fingers to the back and attached to the joint structures at specific points. This transmission approach has advantages in terms of mechanical simplicity and spatial efficiency, which can be used to achieve effective multi-joint coupling in a compact hand configuration.

### 2.2. Mechanical Finger Structure and Coupled Motion

**Figure 2:**
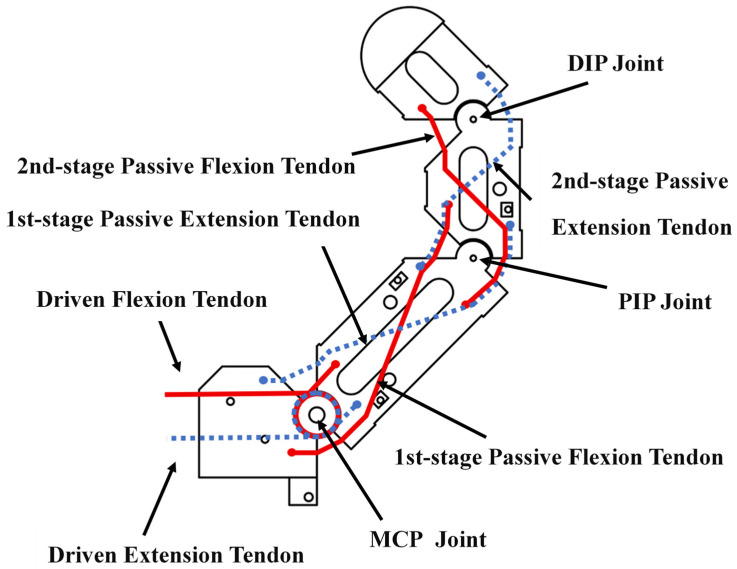
Tendon-routing layout and functional classification of the four-finger module. The figure shows the tendon-routing configuration of a representative finger in the four-finger module, including the driven flexion tendon, driven extension tendon, first-stage passive flexion and extension tendons, and second-stage passive flexion and extension tendons. The MCP, PIP, and DIP joints are also indicated to illustrate the structural basis of the coupled tendon-driven transmission.

As shown in Figure 2, each finger in the four-finger module is organized as a tendon-driven three-joint chain including the MCP, PIP, and DIP joints. To make the routing relationship explicit, the tendon set can be divided into one driven flexion tendon, one driven extension tendon, a first-stage passive flexion/extension tendon pair, and a second-stage passive flexion/extension tendon pair. The driven tendons are directly connected to the motor-side actuation, whereas the passive tendons are arranged to redistribute tendon length during joint rotation and thereby transmit motion across adjacent joints. In the present design, the flexion-side tendons are marked in red, and the extension-side tendons are marked in yellow. For the driven tendon, one end is fixed to the motor-winding drum and the other end is fixed to the proximal phalanx by a screw. For the passive tendon, one end is terminated by a knot-based mechanical stop, and the other end is fixed to the corresponding phalanx in a similar manner. This routing arrangement provides the structural basis for coupled multi-joint transmission under constrained actuation.

**Figure 3:**
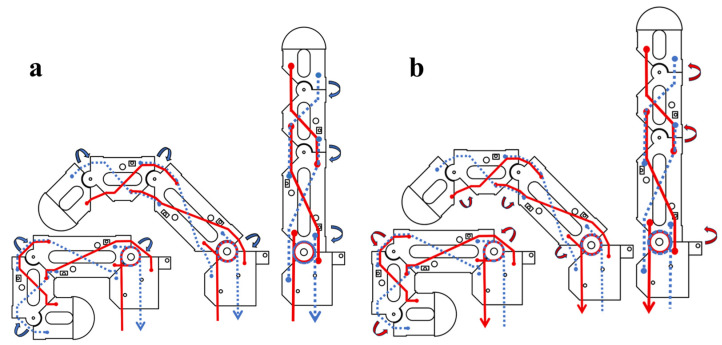
Sequential transmission mechanism of coupled extension and flexion in the four-finger module. (**a**) Tendon-driven transmission during extension. (**b**) Tendon-driven transmission during flexion. The figure illustrates how motor-driven tendon input is first converted into MCP rotation and then further propagated to the PIP and DIP joints through passive tendon coupling.

Figure 3 further illustrates how the tendon-routing arrangement shown in Figure 2 is converted into sequential coupled motion during extension and flexion. In both directions, the motor-driven tendon input first produces rotation at the MCP joint, after which tendon-length redistribution progressively drives the PIP and DIP joints.

During flexion, the driven flexion tendon on the palmar side of the MCP joint is attached to the motor. When the motor rotates forward, this tendon is tensioned, generating a downward pulling force and a counterclockwise driving moment on the proximal, middle, and distal phalanges. Under this moment, the three phalanges initially rotate together about the MCP joint. As the proximal phalanx rotates, the passive tendon segment on the dorsal side of the MCP joint is progressively exposed. Because the total tendon length remains constrained, the tendon length originally assigned to the PIP joint correspondingly decreases, which generates a counterclockwise moment on the middle and distal phalanges and causes them to rotate about the PIP joint. In a similar manner, when the middle phalanx rotates about the PIP joint, the dorsal passive tendon segment at the PIP joint is further exposed, decreasing the tendon length associated with the DIP joint. This produces a counterclockwise moment on the distal phalanx and drives DIP rotation. Therefore, tension applied only to the driven flexion tendon is sufficient to drive the MCP, PIP, and DIP joints sequentially through tendon length–constraint transmission, thereby achieving coordinated multi-joint finger flexion.

For coupled finger extension, the transmission process works in the opposite direction. When the motor rotates backwards, tension is applied to the driven extension tendon on the dorsal side of the MCP joint. Through the same tendon length–constraint relationship, the phalanges rotate in the reverse direction, so that MCP extension is initiated first and the motion is then transmitted successively to the PIP and DIP joints. As shown in Figure 3, the extension process follows the same coupled transmission logic as flexion, but with the tendon-loading direction reversed. Under full coupled motion, each adjacent phalanx rotates by approximately 90° with respect to its neighboring segment, while the passive tendon length is redistributed between the palmar and dorsal sides.

**Figure 4:**
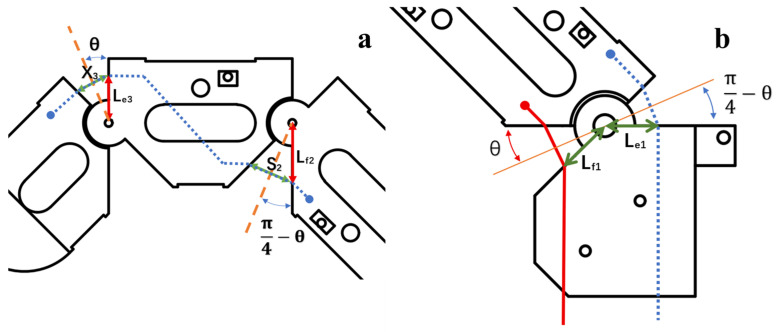
Tendon conditions during finger extension. (**a**) Secondary passive tendon; (**b**) active tendon.

As shown in Figure 4a, taking the secondary passive extension tendon as an example, to keep the tension of the secondary passive extension tendon continuous and the total tendon length constantly equal during the whole motion, it is necessary that the following condition is satisfied:

(1) $$X_{3}\left(\theta =45^{\circ }\right)=S_{2}\left(\theta =0\right)$$

Moreover, since:

(2) $$X_{3}=\sqrt{2}L_{e3}\left(\theta =45^{\circ }\right),S_{2}=\sqrt{2}L_{f2}\left(\theta =0\right),$$

it follows that the passive tendon length should satisfy:

(3) $$L_{e3}=L_{f2}$$

In the designed finger structure, we set:

(4) $$L_{e3}=L_{f2}=5\mathrm{mm}$$

The passive tendon is routed according to this relationship. Experimental results show that this routing strategy is useful to keep the tendon length consistent during coupled motion.

For the active tendon, as shown in Figure 4b, if the same routing configuration as the passive tendon was adopted and the active tendon was connected directly to the motor input, tendon slack may occur in the practical actuation. The main cause of this slack is inconsistency in tendon stroke. Specifically, after a flexion motion, the angle of the dorsal side of the MCP joint increases by 2θ, while the angle of the palmar side of the MCP joint decreases by 2θ. Accordingly, the angle from the top (dorsal) can be written as 2θ and the angle from the bottom (palmar) can be written as:

(5) $$\frac{\pi }{2}-2\theta ,\theta \in \left[0,\frac{\pi }{4}\right]$$

To avoid tendon slack, the active tendon must satisfy stroke consistency during motion:

(6) $$2L_{e1}\sin\theta +2L_{f1}\sin\left(\frac{\pi }{4}-\theta \right)=C$$

However, the above relationship cannot be strictly equal to a constant C. Under this routing scheme, there is not equal stroke of the active tendon during motion, which means that the driving side and the return side have different lengths. In the practical actuation, this stroke mismatch directly compromises the reliability of finger motion.

Specifically, when the motor output torque is low, unequal stroke holds the tendon from staying in effective tension, limiting finger motion or even preventing its initiation. When the motor torque is high enough, the motion can be completed by the finger overcoming the discrepancy of the stroke, but the local tendon slack is introduced, resulting in dead travel, which decreases the accuracy and controllability of the motion.

Therefore, if the active tendon is routed the same as the passive tendon, there is inevitable inconsistency in stroke and stability, and predictability of actuation is compromised. Based on this analysis, the active tendon needs a routing and transmission configuration different from that of the passive tendon to ensure consistent stroke during the driving.

**Figure 5:**
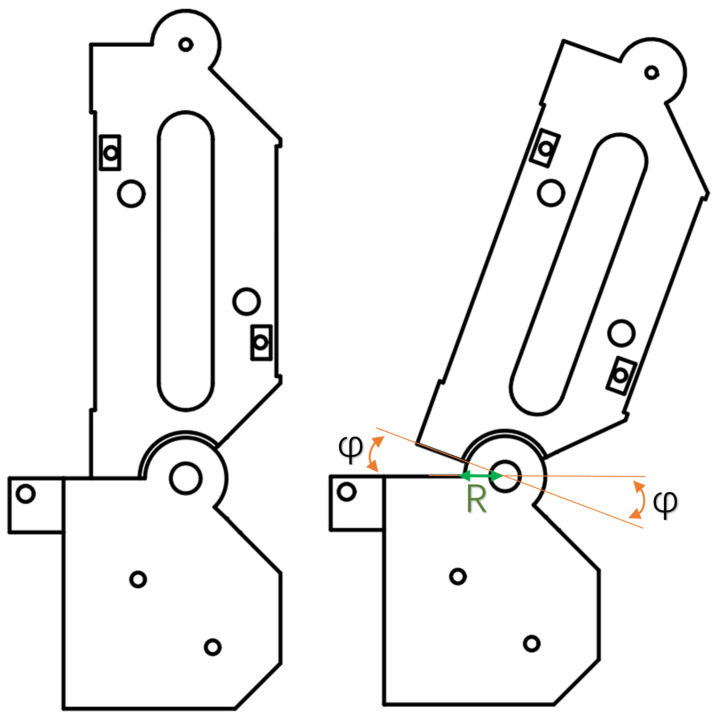
Equal-stroke analysis of the active tendon at the MCP-side transmission interface. The left panel shows the initial aligned state of the active tendon around the MCP pulley, and the right panel shows the rotated state during joint motion. The bearing-guided routing is introduced to maintain coordinated tendon displacement and to avoid unequal stroke during active transmission.

Further analysis of the joint kinematic relationship shows that, as shown in Figure 5, it naturally satisfies the following identity:

(7) $$\phi +\left(\frac{\pi }{2}-\phi \right)=\frac{\pi }{2}$$

Specifically, the sum of the two exposed angles at the joint remains constant at $\frac{\pi }{2}$. Subtracting $\frac{\pi }{2}$ from both sides of this identity and multiplying by R yields:

(8) Rϕ−Rϕ=0

Under this condition, the stroke variations in the tendon segments during motion cancel each other out, to achieve consistency of tendon stroke.

Based on this relationship, the original chord-length variation can be converted into an arc-length variation. By incorporating a bearing into the structure, equal-stroke actuation of the active tendon can be realized. Specifically, when the bending angle is ϕ, the length of the flexion-driving tendon decreases by Rϕ, while the length of the extension tendon increases by Rϕ. These variations are equal in magnitude, satisfying:

(9) Rϕ=Rϕ

**Figure 6:**
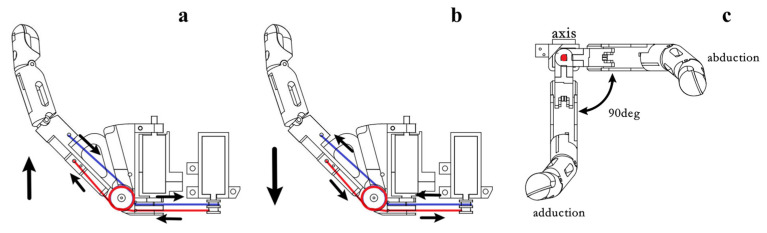
Thumb adduction, abduction, and opposition motions of the proposed thumb module. (**a**) Adduction and (**b**) abduction are realized by differential winding and release of the antagonistic tendons. (**c**) Opposition is driven by the directly coupled motor output. These motions jointly provide the thumb-side configurational adjustment required for representative grasp formation.

In addition to the coupled four-finger mechanism, the thumb adopts a differentiated two-DOF actuation arrangement, as shown in Figure 6. The two independently controlled thumb DOFs are opposition and abduction/adduction, which are deliberately preserved to support grasp-type differentiation at the hand level. For thumb abduction/adduction, two antagonistic tendons are wound and released in opposite directions to generate differential tendon-length variation, thereby generating thumb abduction/adduction motion. For thumb opposition, the motor output shaft is mechanically coupled to the thumb transmission structure, so that the corresponding motion can be produced directly. Through the coordination of these two thumb-side DOFs, the hand retains the configurational adjustment required for representative grasps such as the five-finger power grasp, tripod pinch, and lateral pinch.

### 2.3. Control Architecture and Strategy

#### 2.3.1. Overview of the Control Architecture

**Figure 7:**
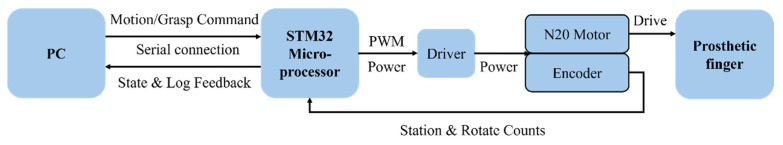
Overall architecture and data-flow organization of the control system.

To facilitate the coordinated use of more than one finger and ensure the successful execution of grasps, this work implements a layered control architecture, as shown in Figure 7. The system consists of a host personal computer (PC) and an embedded control unit (responsible for command interaction and low-level execution, respectively). The control pipeline is a closed-loop data flow which includes “command transmission → execution → state feedback.”

The host PC acts as the interaction interface at the upper level, generating and trans-mitting predefined commands for hand postures and grasp modes, and controlling the serial communication with the embedded controller. Motion commands are sent to the lower-level controller over a serial link and runtime status and feedbacks from the embedded unit are returned to the PC in real-time for experimental monitoring and data logging.

The embedded control unit is based around an STM32F767 microcontroller. It receives and parses commands from the PC and generates motor drive signals and coordinates the execution process in a unified way. The microcontroller directly interfaces with the drive motors and their encoders and continuously receives encoder feedback to characterize the states of the motor motion and provide essential inputs for the subsequent control logic and state determination.

Control signals are distributed by the STM32 to individual motor driver modules, which execute their assigned command independently of each other. Encoder feedback is gathered and aggregated back to the control unit, creating a centralized state acquisition path for multi-motor coordination. This data-flow organization eliminates cross-module coupling dependencies, which allows for a consistent interface even as motor channels are scaled up or control logic is altered.

The hardware implementation is based on the actuation of the motor and the acquisition of position feedback. All the information needed for control and state determination is measurable by the encoders and processed centrally by the embedded unit, thus offering a complete signal chain for implementing the further control strategy. In the present implementation, command generation and motion execution are organized in a command-driven manner, whereas encoder feedback is used primarily for execution-state monitoring, contact/near-stall inference, and event-triggered termination of individual motors.

#### 2.3.2. Control Strategy

**Figure 8:**
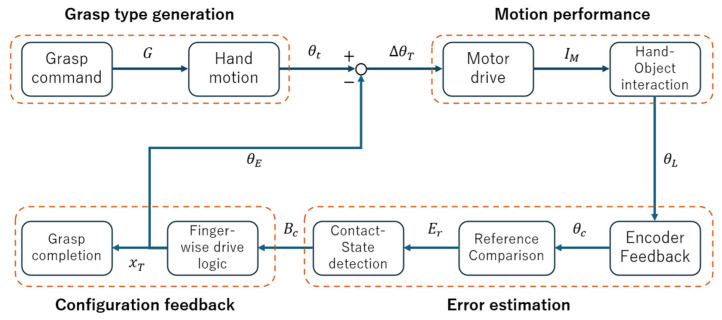
Control block diagram of the proposed grasp execution strategy. It shows the forward command–execution path and the encoder-informed supervisory feedback path that performs reference comparison, contact/state detection, and finger-wise motor-stop decisions for grasp completion and overdrive prevention. Here, G denotes the grasp command, $\theta_{t}$ the target state, $\Delta \theta_{T}$ the resulting drive increment after feedback correction, $I_{M}$ the motor-drive signal, $\theta_{L}$ the interaction-related motion state, $\theta_{c}$ the encoder-measured state, $E_{r}$ the reference-comparison error, $B_{c}$ the detected contact/state condition, $x_{T}$ the termination signal, and $\theta_{E}$ the feedback term returned to the forward path.

Figure 8 presents the control block diagram of the proposed grasp execution strategy, which is organized as two coordinated paths: a forward command-execution path from grasp command to motor drive and hand–object interaction, and an encoder-informed feedback path involving reference comparison, contact/state detection, and per-finger stop logic for grasp completion and overdrive prevention. During the system calibration stage, repetitive no-load trials are performed to characterize the stable rotational behavior of each motor under the corresponding motion commands. This process sets a range of reference rotation for each motor in the absence of external disturbances. The reference range is then used in the control workflow to determine the joint behavior that is expected with ideal motion conditions to provide the foundation for the reference speed model $\omega_{\mathrm{ref}}\left(t\right)$ and reference rotation range ${\mathrm{range}}_{\mathrm{ref}}$ for the free motion phase. On this basis, the execution process is organized into a free-motion state, in which motor behavior is expected to remain close to the calibrated reference, and a contact-affected state, in which deviations from the reference are used to trigger termination decisions. Accordingly, the forward path is responsible for carrying out the commanded grasp motion, whereas the feedback path supervises state transitions relative to the calibrated reference and determines whether motor-specific intervention is required.

During operation, the embedded controller actuates the designated motors according to commands it receives while continuously sampling encoder feedback to estimate the motor angular velocity $\omega_{i}\left(t\right)$. Regardless of the engagement of the fingers, the rotational state of each motor is constantly updated under encoder feedback constraints.

During free motion, encoder feedback remains close to the calibrated reference behavior, and the corresponding motors continue their commanded rotation toward the reference rotation range. Once external contact restricts joint motion, the motor rotational state departs from the reference behavior. In the present strategy, this deviation is used not for continuous force regulation, but for execution-state inference; in particular, it appears as a pronounced decay of $\omega_{i}\left(t\right)$ relative to $\omega_{\mathrm{ref}}\left(t\right)$ and serves as the main basis for contact-related termination decisions. Accordingly, the proposed strategy should be understood not as purely open-loop execution, nor as continuous closed-loop regulation of grasp interaction variables, but as command-driven grasp execution under encoder-informed supervisory feedback.

Based on these observations, the embedded controller calculates contact states by observing motor speed patterns. When the rotational speed decreases quickly within a short period of time and becomes close to zero, the finger is said to be rigidly blocked. This criterion may be written as:

(10) $$\exists t^{*}\in \Delta t:\omega_{i}\left(t^{*}\right)=0,\;\frac{d\omega_{i}\left(t\right)}{dt}\ll 0$$

In this case, in an established time interval, the rotational velocity is much slower than during the free-motion period but not equal to zero, and the finger is said to be in contact with a deformable body. This criterion may be stated as follows:

(11) $$\omega_{i}\left(t\right)<k\;\omega_{\mathrm{ref}}\left(t\right),\;\omega_{i}\left(t\right)>\omega_{min}>0$$

where $k\in \left(0,1\right)$ denotes the relative drop threshold and $\omega_{min}$ represents the lower bound for a non-zero speed. In both cases, the drive output of the corresponding motor is terminated, and the current rotational state is recorded.

During coordinated multi-motor operation, the state of each motor is determined independently. Once a motor reaches its reference rotation range or a contact decision is triggered, its actuation is stopped, while the others keep on going according to their respective rotational states. This approach allows a coordinated multi-finger closing process that automatically adapts to different contact conditions. Accordingly, the coordinated grasping process is not governed by a single global stopping condition or by continuous interaction-variable regulation, but by encoder-informed supervisory feedback with event-triggered termination at the level of individual motor channels.

## 3. Results

### 3.1. Basic Performance Characterization of the Prosthetic Hand

After the structural design of the prosthetic hand was completed, all the components were made and assembled based on the predefined mechanical scheme. The overall hand structure was manufactured by fused deposition modeling additive manufacturing. The phalangeal links, palm shell and supporting components were printed in carbon-fiber-reinforced polylactic acid to provide a balance between structural stiffness and overall weight reduction.

Following printing, the structural parts were subject to dimensional finishing, prior to assembly. The PIP and DIP joints were realized as revolute joints with pin joints and miniature bearings were used at the MCP joints to minimize rotational friction and increase motion stability. These joint designs meet the requirements for repeated flexion and extension under the actuation of a tendon.

During assembly, the fingers were mounted sequentially onto the palm body followed by the connection and initial tensioning of the drive motors and tendons. The thumb used an independent dual-actuation configuration with the rotational and translational DOFs driven by the corresponding motors and calibrated during assembly. The overall assembly process was organized by functional modules, which facilitates local adjustment and maintenance.

**Figure 9:**
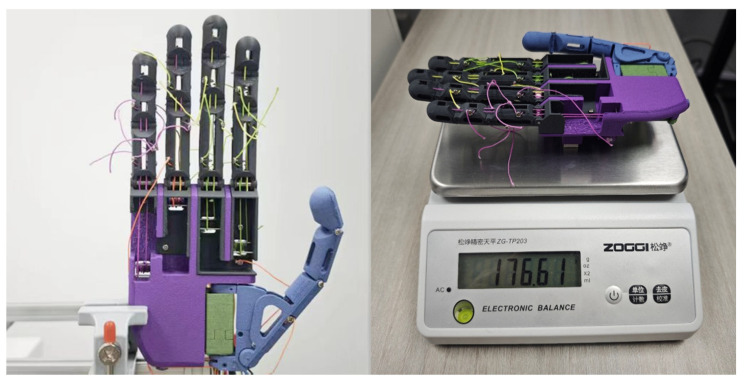
Photograph of the proposed prosthetic hand prototype.

The assembled prosthetic hand is shown in Figure 9. With all drive motors, tendon transmission components and structural parts integrated, the total mass of the hand is 176.6 g. The maximum force output of a single finger was measured by a quasi-static test with the load applied to the fingertip pad resulting in a peak force of about 2.8 N. The test protocol was similar to the later grasping experiments, and the actuation and control parameters were kept the same. These results provide basic mechanical reference information for the subsequent posture-reproduction and grasp-formation experiments.

### 3.2. Reproduction of Representative Hand Postures

This section uses the GRASP taxonomy proposed by Feix et al. as the organizational and evaluation framework for the grasp action library [44]. The taxonomy divides the typical human grasps into 33 types and describes the configuration differences in multiple dimensions, such as grasp family (Power/Intermediate/Precision), thumb posture (abducted/adducted), and opposition/contact form (palm/pad/side).

**Figure 10:**
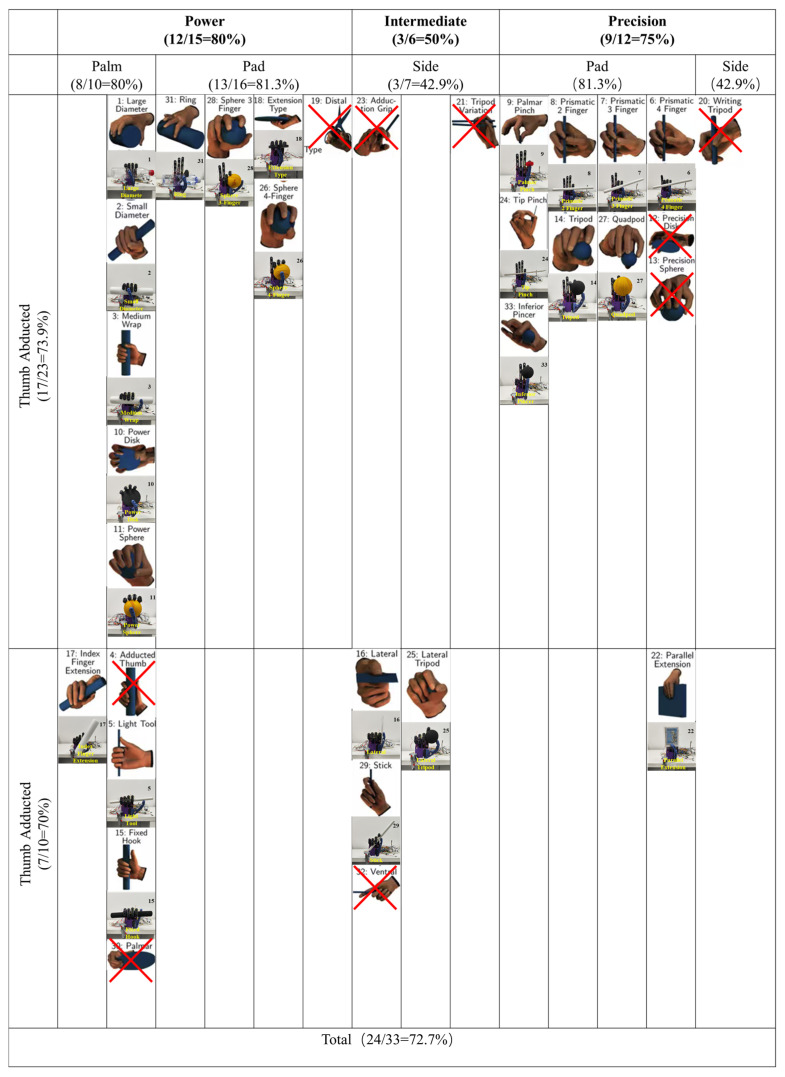
Mapping of reproduced grasp types and taxonomy-level coverage based on the Feix GRASP taxonomy. Each grasp type is displayed at its corresponding taxonomy position. For reproduced grasp types, both the original illustration from the Feix taxonomy and the corresponding prototype demonstration are shown, while the grasp types not achieved by the proposed prosthetic hand are marked with red crosses. The reproduced quantity and proportion of each major grasp category are also indicated in the figure.

Using this framework, the set of grasps the proposed prosthetic hand can achieve is mapped onto the taxonomy grid, with representative objects being chosen for configuration-level verification (Figure 10). Figure 10 further summarizes the taxonomy-level coverage and its distribution among grasp families, thumb posture, and opposition/contact forms, thereby characterizing both the extent and structural profile of the reproduced grasp repertoire.

Using the GRASP taxonomy proposed by Feix et al. as a reference, the reproduced grasp set of the proposed prosthetic hand (Figure 10) is mapped onto the taxonomy grid, with a physical example provided for each grasp type. The reproduced set covers all three grasp families, Power, Intermediate and Precision, and shows cross-category distributions with respect to thumb posture and opposition/contact form. The overall coverage and distribution statistics are indicated in Figure 10.

In the experiments, the prosthetic hand was started from a fully open posture and was closed according to predefined control commands to contact the target objects. As shown in Figure 10, different grasp types showed stable and distinguishable differences in thumb opposition, four-finger participation patterns, primary contact regions (palm surface, fingertip pad, or finger side), and overall configuration geometry, forming clear separable functional configurations.

As shown in Figure 10, compared to the Feix GRASP taxonomy, the proposed system can reproduce 24 out of 33 canonical grasp types. The reproduced types represent all three grasp families, Power, Intermediate, and Precision, both abducted and adducted thumb postures, as well as palm, pad, and side opposition/contact forms. This distribution shows that the prosthetic hand can switch between palm-based envelopment, fingertip-pad pinching, and lateral clamping, and thus allows an action library representation to exhibit functional discriminability.

This structure of coverage corresponds to the system’s structure—control configuration. For the four fingers, active MCP actuation allows the main closing stroke and output, and underactuated coupling of the PIP/DIP joints allows for compliant flexion under con-tact constraints, which allows for continuous contact formation and stable enveloping power grasps. Meanwhile, the thumb’s two independently active DOFs allow for adequate posture-adjustment space for opposition-point placement and the pinching plane, allowing for precision and intermediate grasps that are dominated by pad or side contact. Overall, the “palm-pad-side” cross-mode coverage as seen in the taxonomy-level distribution reflects a synergistic interaction of underactuated envelopment capability and thumb-posture adjustability, which supports the expression of diverse grasp configurations.

The following section selects representative grasp families and object categories from the reproduced set to further analyze contact formation, per-finger termination behavior, and configuration adaptation under different geometric conditions and limited loading variation.

### 3.3. Grasping Experiments

Building on the action library reproduction results based on the GRASP taxonomy, this section picks representative grasp families for task-level validation and mechanistic analysis. The focus is on (i) the contact-formation process induced by the underactuated structure under object constraints, and (ii) per-finger motor-stop behavior and configuration convergence characteristics triggered by the encoder-feedback-based termination strategy under varying contact conditions. The experiments cover three types of grasp modes: five-finger power grasp, tripod pinch, and lateral pinch. The five-finger grasp is an enveloping power grasp and is used to investigate the formation of multi-point contact and enclosure stability on objects with regular and complex geometry. The tripod pinch tests the thumb–index–middle opposition configuration and the consistency of the convergence of the system, and the lateral pinch tests the system’s ability to form a stable clamping plane when grasping thin, plate-like objects. Grasped objects are stratified in terms of geometry, size, and weight, summarized in Table 4.

During the experiments, the prosthetic hand was initialized from a uniform fully open posture, and predefined grasp commands were issued by the host PC. The embedded controller performed the closing motion using encoder feedback and terminated each motor when a contact or near-stall condition was detected using the speed-decay criterion. The results were mainly analyzed in terms of final post-grasp configuration, contact distribution characteristics, and configuration consistency on different objects with the objective of understanding the grasp formation mechanism resulting from the coupled effects of underactuated structural coupling, contact constraints, and per-finger termination.

#### 3.3.1. Geometric Adaptability in Five-Finger Power Grasp

**Figure 11:**
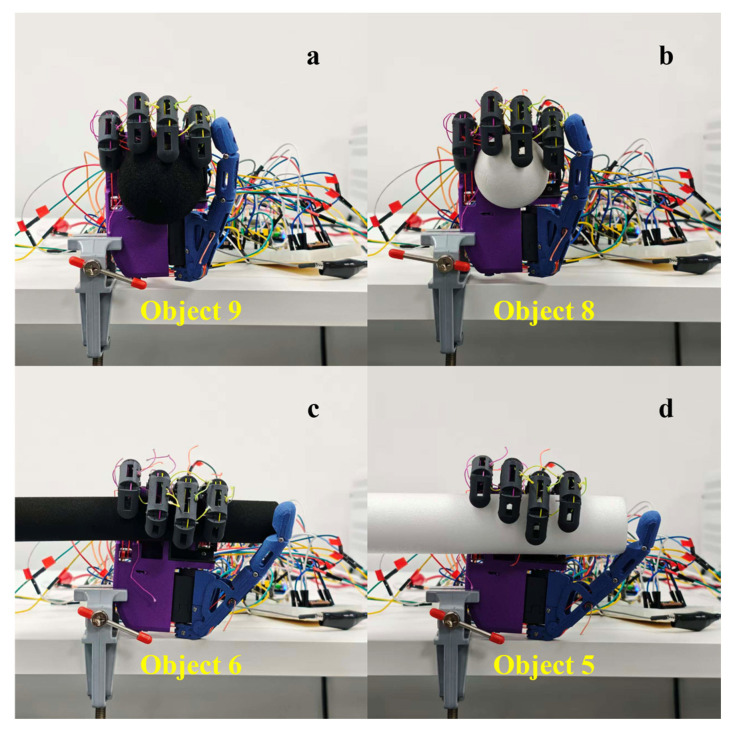
Five-finger power grasp configurations on regular geometric objects. (**a**) Medium sphere; (**b**) small sphere; (**c**) small cylinder; (**d**) large cylinder.

**Figure 12:**
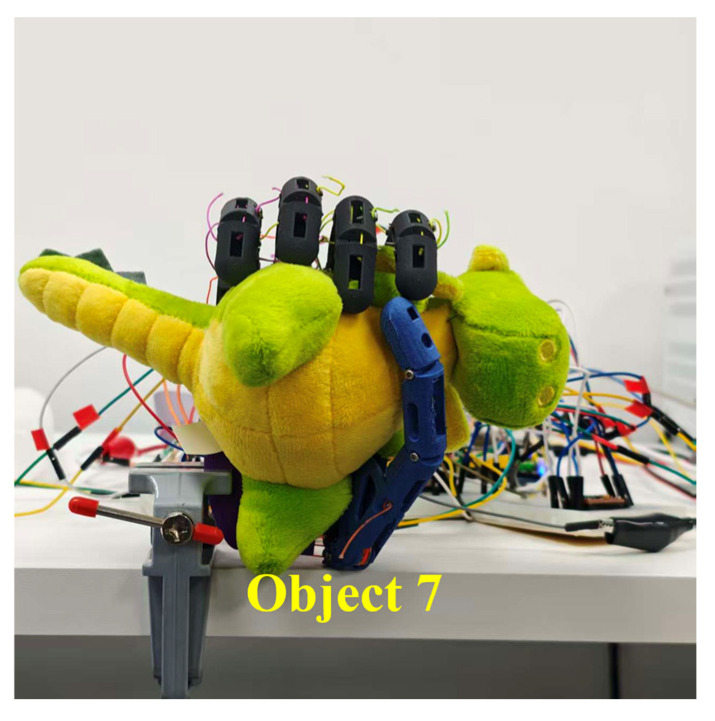
Five-finger power grasp performance on a complex-geometry object.

The five-finger power grasp was designed to study the configuration convergence behavior of underactuated enveloping grasps over objects of different geometries. Two spheres and two cylinders were chosen to represent regular geometries with the corresponding grasp configurations illustrated in Figure 11. A plush toy was further used as a complex-geometry object for validation where the result is shown in Figure 12. Motors 1–4 correspond to the little, ring, middle and index fingers, respectively, while motors 5 and 6 correspond to the flexion/extension and adduction/abduction DOFs of the thumb.

For regular geometries, the five-finger grasp configuration consistent closure adjustment according to the target shape is exhibited, as shown in Figure 11. For the spherical object, a clear relationship of thumb and four-finger opposition is formed, with contact being distributed mainly over the pads of the fingertips and the area of palmar envelopment. For the cylindrical object, the contact band is along the axial direction, and the contribution of the four-finger envelopment is more pronounced.

For the complex-geometry object (plush toy), Figure 12 reveals a very asymmetric distribution of contact points in which local protrusions and soft-tissue deformation cause finger-specific variations in closure depth. This indicates that under irregular boundary conditions the combined effects of underactuated coupling and per-finger termination drive the fingers to converge to differentiated stop positions at different contact instants, thus forming an enveloping configuration which is stable.

#### 3.3.2. Configuration Consistency Under Limited Loading Variation in Five-Finger Power Grasp

**Figure 13:**
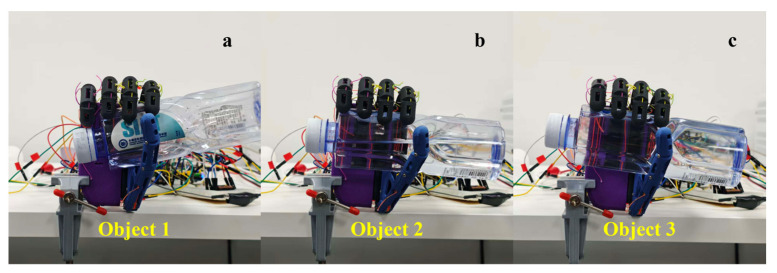
Five-finger power grasp performance under different load conditions. (**a**) Light bottle; (**b**) medium bottle; (**c**) heavy bottle.

To assess the consistency of grasp-configuration formation under constant geometric constraints and limited loading variation, a plastic water bottle was used with three different water levels to achieve varying weights, defined as the light bottle, medium bottle, and heavy bottle, respectively. Grasp tests were performed under these three loading conditions. The results can be seen in Figure 13. In these experiments, grasp termination was accomplished using encoder-feedback-triggered, per-finger independent decisions, in which the closing process for each finger terminated when either the reference stroke was reached or when a speed decay contact signature was detected. When the object geometry was kept constant, the geometric contact formation and the resulting kinematic constraints remained the dominant factors determining the termination instant, whereas the tested loading variation did not alter the overall configuration outcome.

As shown in Figure 13, for the three weight conditions, the overall enveloping morphology and relative finger-to-finger positions are consistent with no visible re-distribution of contact. This suggests that, under the combined constraints of the underactuated structure and the encoder-feedback-based termination mechanism, grasp-configuration convergence is mainly governed by geometric contact conditions. Within the tested range, the loading variation did not produce a significant shift in the overall grasp configuration.

#### 3.3.3. Opposition and Lateral Clamping Capability in Precision Grasps

**Figure 14:**
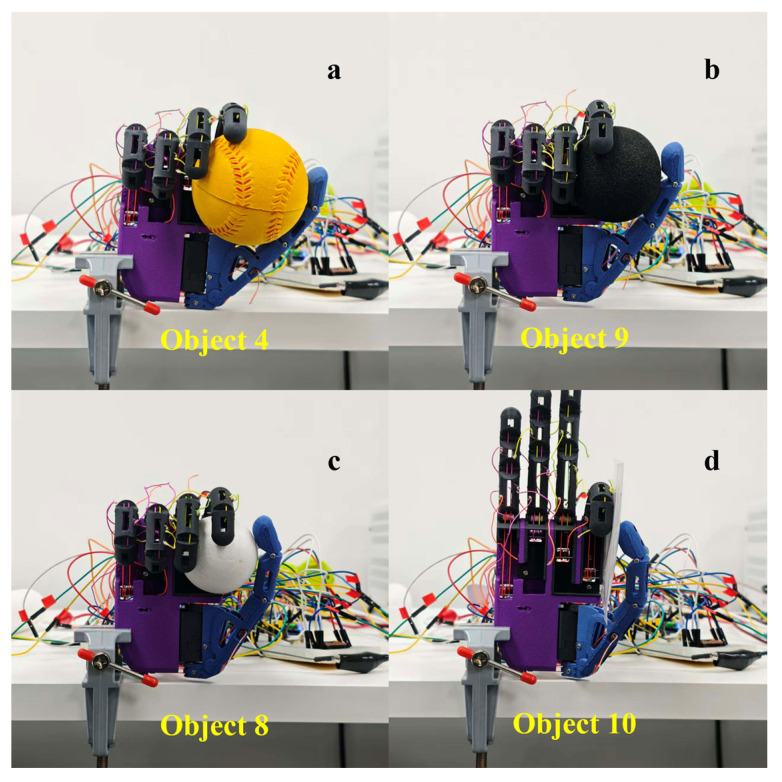
Multi-finger precision grasp configurations in fine manipulation tasks. (**a**) Tripod pinch on a large sphere; (**b**) tripod pinch on a medium sphere; (**c**) tripod pinch on a small sphere; (**d**) lateral pinch on a card.

Precision grasping tasks mainly test the ability of the thumb and long fingers to obtain accurate opposition positioning on small-scale targets and to form an effective clamping plane. In this study, precision grasping performance was assessed by tripod pinch and lateral pinch tasks (Figure 14).

The tripod pinch is based on stable pad-to-pad opposition between the thumb and index/middle fingers. Figure 14a–c indicate that, for different-sized spheres, the relative finger positions make subtle changes while preserving a clear opposition geometry. As the target size is increased, the distance of opposition and the location of clamping is rearranged to maintain a stable pinch. The rotation of non-participating fingers is low, focusing actuation on the fingers necessary for opposition and thereby improving repeatability of configuration formation.

The lateral pinch focuses on the formation of a lateral clamping plane on thin, plate-like targets. As can be seen in Figure 14d, the card is clamped stably between the thumb and the lateral surface of the index finger, resulting in a well-defined geometric constraint between the clamping line and the card plane. These experiments show that the dual-DOF thumb configuration allows for both pad-to-pad opposition and lateral clamping of thin objects and supports a variety of precision-grasping capabilities.

## 4. Discussion

This paper is dedicated to lightweight design and engineering feasibility and presents a multi-DOF biomimetic prosthetic hand driven by six motors. Its functional performance is systematically validated by experiments on hand-posture reproduction and grasping. Unlike multi-finger hands that mainly seek a high DOF or rely on a complex sensor-based closed-loop control, the present work focuses on effective coverage of daily functional hand states under constraints on the number of actuators and system complexity, by means of a deliberate integration of the mechanical structure and control strategy. The following discussion is organized according to the experimental findings, with special emphasis on posture reproduction, grasp-formation mechanisms and comparisons with existing prosthetic-hand systems.

### 4.1. Relationship Between Posture-Reproduction Results and Joint-Coupling Structure

A key question in posture reproduction is whether, given a limited actuation budget, the system will still be able to achieve a sufficiently rich set of functionally distinguishable hand configurations. Within the GRASP taxonomy framework proposed by Feix et al., the proposed prosthetic hand spans the three grasp families (Power, Intermediate, and Precision), cross-category distributions in thumb posture (abducted/adducted) and opposition/contact form (palm/pad/side). Rather than reducing to a single enveloping closure pattern, the resulting repertoire covers multiple grasp configurations with differences in opposition location, contact organization, and finger participation.

This level of coverage is closely related to the design of the joint coupling architecture. Prior studies of synergies and posture statistics of human hands have suggested that functional hand states in daily activities tend to be clustered around a few low-dimensional synergy modes, which may be well represented by a limited number of control variables [45,46,47,48]. The adopted organization embeds part of these synergy relationships mechanically: the MCP joint provides the primary closing stroke and energy input, while the PIP/DIP joints allow contact-dependent flexion allocation under the combined effects of tendon-length constraints and external contact forces. As a result, multiple stable grasp configurations remain reachable under low-dimensional inputs. The simultaneous presence of Power and Precision grasps in the taxonomy indicates that the proposed architecture preserves differentiated closure behaviors under object constraints: Power grasps are associated with multi-finger envelopment and sustained contact formation, whereas Precision grasps depend on localized opposition and contact-region organization. Compliance induced by underactuated coupling after contact promotes robust formation of enveloping configurations across object geometries, whereas finer opposition and clamping configurations demand higher demands on thumb-posture adjustment.

In this context, the two-DOF thumb configuration plays a key role in grasp-type differentiation. Thumb adduction and pronation allow for adjustable DOF in opposition-point placement and clam-plane formation, which allows the system to implement stable constraints in palm-dominated enveloping grasps, as well as to realize pad-dominated pad-to-pad pinches and side-dominated lateral clamps. Accordingly, cross-dimensional coverage in the GRASP taxonomy with respect to thumb posture and opposition form can be seen as a direct manifestation of the combined effect of “thumb posture DOFs + four-finger underactuated envelopment”: the long fingers deliver sustained closure and envelopment capability, and the thumb delivers the geometric adjustment space needed for configuration differentiation. Together, they determine the reachable range and distribution of grasp types.

Compared with fully independently actuated multi-finger hands, such a coupled architecture has inherent limitations in joint-level fine control. Its advantage, however, is in embedding synergy relationships directly into the mechanism and tendon-routing design, which decreases the sensitivity of configuration formation to control inputs and allows for stable and functionally distinguishable configurations. Furthermore, compared to highly underactuated single-input synergistic structures (e.g., the SoftHand Pro), the proposed underactuation scheme keeps the number of actuators low while increasing the freedom of thumb-related configurational freedom, enabling opposition and lateral-clamping configurations to be expressed more explicitly within the repertoire [47]. Overall, these structural choices translate the “low-dimensional synergy” principle of human hand function into an engineering-realizable configuration boundary: a high proportion of canonical grasp types is achieved with a limited actuation scale, while also providing a clear structural basis for expanding grasp types or incorporating higher-level control strategies within the same mechanical framework.

### 4.2. Grasping Results and Structure–Control Synergy

In the grasping experiments, the prosthetic hand was able to achieve stable grasps on regular objects (spheres and cylinders), irregular objects, and thin plate-like objects under representative modes, including the five-finger power grasp, tripod pinch, and lateral pinch. Variations in object size, shape, and weight were dealt with mostly by self-adjustments in multi-finger envelopment and contact-point distribution, and not by fine joint-level force regulation.

From the grasp category point of view, the results of research on the classification of the human hand and daily manipulation show that the five-finger power grasp, the tripod pinch and the lateral pinch are among the most common patterns in daily life and are widely used as bench-mark modes in the evaluation of prosthetic hands [44,49,50,51]. Accordingly, the current experiments were designed around these three modes and show that even with limited actuation, stable grasp configurations can be achieved through coordinated multi-finger envelopment and underactuated joint coupling.

Structurally, the relatively high overall DOFs and multi-finger coordination allow phalangeal postures to be passively adjusted after contact to accommodate geometric variations. Building on this mechanically embodied adaptability, the control strategy mainly manages the grasping process and performs state determination. Grasp termination is initiated by encoder-derived changes in motor rotation, recognizing motion-constrained states following contact and preventing mechanical loading of the system due to continued drive. While not designed to allow fine-grained force modulation, this strategy is complementary to the underactuated envelopment capability, allowing robust grasp stability in limited sensing conditions. The present evaluation focuses primarily on grasp-type coverage, configuration formation, and grasp-behavior consistency under constrained actuation and sensing. Although the measured peak single-finger driving force provides a basic indication of local output capability, it does not constitute a direct quantification of whole-hand grasp force, payload, or load-dependent grasp success. Accordingly, the current results are more appropriately interpreted as evidence of functional grasp formation under lightweight constraints than as a complete characterization of strength-related performance. More systematic evaluation of pinch force, whole-hand grasp force, and payload-dependent success remains an important direction for future work.

### 4.3. Comparison with Representative Prosthetic-Hand Systems

**Figure 15:**
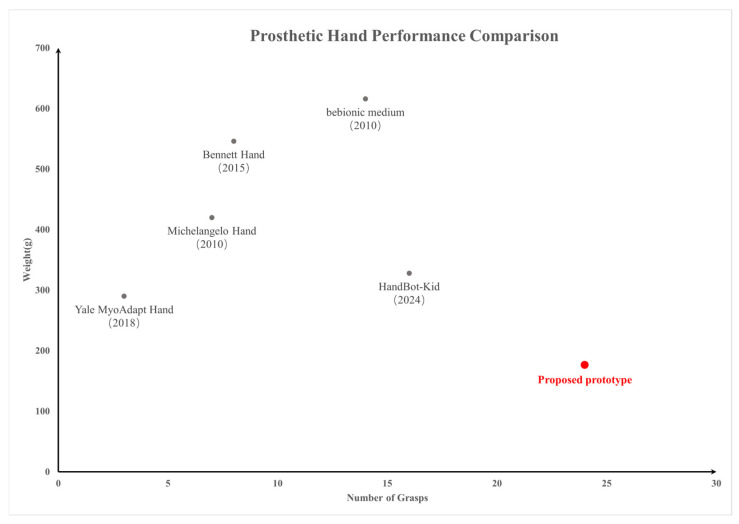
Comparative positioning of representative prosthetic hands in the weight–grasp repertoire space. The horizontal axis denotes the reported number of representative grasps, grasp types, or grasp/posture patterns in the corresponding sources, and the vertical axis denotes hand weight. The proposed prototype is shown using the current hand body mass. Black dots represent the representative prosthetic hands used for comparison, and red dots represent the proposed prosthetic hand.

A comparison among current prosthetic-hand design routes emerges when shifting the evaluation from structural descriptors alone to the relation between wearable mass and retained grasp repertoire. In this context, the proposed prototype is compared with representative research and commercial prosthetic hands in a weight–grasp space, as shown in Figure 15. The selected examples cover lightweight research platforms and established multi-grasp commercial systems, including the Yale MyoAdapt Hand [41], the Bennett Hand [43], the bebionic medium [52], HandBot-Kid [53], and the Michelangelo Hand [54]. Within this comparison, the more informative distinction lies in how representative grasp differentiation is retained under different mass budgets and design allocations, rather than in actuator number or nominal degrees of freedom alone.

The distribution in Figure 15 suggests that existing systems generally follow different trade-off paths. Some research-oriented hands maintain relatively low mass but support only a limited number of grasp patterns, as in the Yale MyoAdapt Hand [41], whereas other designs expand grasp provision at the cost of markedly increased system mass, as represented by the Bennett Hand [43] and the bebionic medium [52]. Commercial multi-grasp systems such as the Michelangelo Hand and bebionic provide mature grasp repertoires, but this is typically accompanied by a heavier integrated hand-level package. HandBot-Kid occupies an intermediate position, showing that increased grasp diversity can also be approached within a lighter platform, although under a different structural scale and application background. Against this backdrop, the proposed prototype remains in a distinctly lower-mass region while retaining a comparatively broader reported grasp repertoire.

This positioning is consistent with the design logic introduced in Section 1. Recent studies have enlarged prosthetic hand capability through substantially different routes, including increasing articulated mechanical complexity, reducing user burden by multimodal sensing and semi-autonomous execution, or uniformly simplifying the hand toward basic opening–closing functions or a small set of preset patterns. The present study adopts a different allocation strategy. Mechanical simplification is concentrated on the four-finger side through tendon-coupled underactuated closure, while thumb-side geometric adjustment is deliberately retained as the principal source of grasp-type differentiation. This differentiated allocation allows representative precision and conformal grasps to be preserved without extending actuation count or introducing richer sensing loops, which is also in line with prior observations that thumb orientation and actuation play a decisive role in the realization of representative grasp forms, especially tripod and lateral grasps, and that selectively differentiated drive allocation can be more effective than uniform simplification when both grasp diversity and structural compactness must be maintained.

It should also be noted that the reported mass of the proposed prototype refers to the hand body itself. At the present stage, the system has not yet incorporated wrist components, a coupling chamber, battery packaging, or full wearable integration. In contrast, the reference systems used for comparison are largely reported as more integrated hand-level products or prototypes, and their masses therefore reflect a broader hardware scope. The present comparison should thus be interpreted as a hand-level design positioning focused on the relation between intrinsic hand mass and retained grasp repertoire, rather than as a fully system-equivalent evaluation. Even with this boundary acknowledged, the current hand body accounts for only 176.6 g, leaving substantial mass margin for subsequent integration.

Taken together, the distinction of the proposed hand lies not simply in achieving a lower weight or a larger number of grasp patterns in isolation, but in how these two attributes are obtained simultaneously. The contribution of this work is therefore better understood as a selective under-actuation route for retaining representative grasp diversity in a lightweight, sensor-minimal prosthetic hand, rather than as a direct pursuit of maximum dexterity or minimum mass alone.

### 4.4. System-Level Considerations for Future Development

The present study focuses on the hand-level design and validation of a lightweight multi-finger prosthetic hand, with emphasis on actuation allocation, tendon-driven underactuated transmission, and encoder-based grasp execution under constrained hardware conditions. In subsequent development, the hand will need to be extended from a laboratory prototype to a wearable prosthetic system through further system-level integration. At the same time, the compact hand-level architecture was chosen to leave room for subsequent integration of wrist, socket-side, and packaging components at the system level.

This transition from prototype validation to system-level deployment also requires a broader interpretation of performance beyond the current hand-level evidence. At present, the reported results mainly verify representative grasp formation, configuration consistency, and execution stability under lightweight and sensor-minimal conditions. In this context, the measured peak single-finger output should be regarded as a localized indication of actuator-side capability rather than a complete descriptor of practical grasp strength at the system level, which further depends on contact distribution, inter-finger coordination, object properties, and loading conditions during grasping. Accordingly, metrics such as pinch force, whole-hand grasp force, payload capacity, and load-dependent grasp robustness should be considered important targets in the next stage of system-level evaluation.

One important direction concerns onboard power supply. In a wearable implementation, battery placement will need to be considered together with mass distribution, distal inertia, and wearing comfort. Rather than integrating the battery entirely within the hand, a more practical solution is to coordinate battery location with the forearm, wrist, or socket-side layout, so that power supply, packaging, and overall weight distribution can be addressed jointly. Under this system-level design framework, the final battery configuration and its associated mass will be determined together with the required operating duration and overall wearable architecture. Accordingly, battery mass is not reported here as a fixed hand-level parameter.

Further development from the present hand-level prototype toward a wearable prosthetic system will require staged progression. The first step is system-level integration, including the socket interface, wrist module, and complete electromechanical packaging. On this basis, the next step is user-oriented validation, which includes user-specific fitting, long-term durability, and safety assessment under repeated use conditions. A further stage concerns eventual translation beyond the laboratory setting, including the engineering and evaluation work needed for clinical deployment and regulatory compliance. Together, these steps define the main development path from the current prototype to a practically deployable prosthetic system.

## 5. Conclusions

This work examines the structural design, transmission organization, and sensor-free control strategy for a lightweight, underactuated multi-finger prosthetic hand, and presents a prototype featuring 14 joint DOFs using six miniature motors. Structurally, the four fingers use active MCP actuation coupled with underactuated PIP/DIP coupling, while the thumb has two active DOFs to increase the opposition capability and expand the posture-adjustment workspace. At the transmission level, length constraint relationships between active and passive tendons are constructed, and an equal stroke actuation structure is proposed to reduce tendon slack and stroke inconsistency, improving the stability of multi-joint synergistic flexion. At the control level, a layered architecture based on the STM32F767 microcontroller and encoder feedback is developed, as well as a grasp termination strategy using a combination of reference rotation range constraints and speed decay-based contact detection. This approach provides independent per-motor termination, overdrive protection and reliable grasp formation without the need for force or tactile sensing.

Experimental results show that the total mass of the proposed prosthetic hand is 176.6 g and the peak single-finger driving force is about 2.8 N. Regarding grasp function reproduction, using the GRASP taxonomy proposed by Feix et al., the prototype grasps successfully reproduce all three grasp families (Power, Intermediate, and Precision) and span the key configuration dimensions, including thumb abduction/adduction and palm–pad–side opposition/contact forms. These findings indicate the ability of the system to accomplish action library-level functional coverage, under a limited actuation budget. In task-level validation, the hand successfully performs three representative grasp modes, the five-finger power grasp, tripod pinch and lateral pinch, while forming stable grasp configurations on objects with diverse geometries. Regular objects are grasped with continuous envelopment and distributed multi-point contacts; complex objects give asymmetric but stable adaptive contact distributions; and for geometrically identical objects under different loading, the grasp configuration is consistent. Overall, without the need for complex sensing subsystems such as force or tactile sensors, this study shows an interpretable synergy between underactuated structural organization, equal-stroke tendon transmission and encoder-feedback-based termination control. The proposed framework establishes a reproducible engineering route for coordinating structural allocation, tendon transmission, and grasp-termination control in lightweight multi-finger prosthetic hands and supports the implementation of broad grasp-type coverage under constrained actuation and sensing. The reported results consistently support a functional organization composed of coordinated four-finger closure, post-contact adaptive redistribution, and thumb-mediated grasp differentiation. These results indicate that, under simultaneous constraints on weight, actuation, and sensing, grasp diversity is influenced not only by mechanical allocation across digits, but also by how the resulting hand is executed and stabilized. In the present design, the retention of thumb-side geometric adjustment, together with selective underactuation of the four fingers and encoder-informed sensor-minimal execution, forms the basis for preserving representative grasp differentiation. More broadly, the study suggests that, for lightweight prosthetic hands operating under limited sensing, preserving representative grasp differentiation depends less on maximizing articulation everywhere than on selectively retaining the structural and execution-related factors that most strongly shape opposition and grasp completion.

It should be noted that the proposed control strategy focuses primarily on reliable termination of the grasp and overdrive prevention and does not yet provide closed-loop regulation of the grasp force, slip or fine manipulation. Instead, encoder feedback is used here to support execution-state monitoring and event-triggered motor termination during grasp formation under underactuated contact conditions. Additionally, because of underactuated coupling and nonlinearities associated with the tendons, accurate joint-level angle measurement and error quantification need to be further improved. Future work will focus on advancing wearable integration and supporting more demanding tasks by establishing joint-level repeatability metrics using vision-based methods or higher-resolution observation as well as exploring information fusion, such as combining motor-current signatures with simplified tactile sensing, to enhance grasp stability and fine manipulation capabilities without substantially increasing system complexity. These developments will be followed by validation in dynamic tasks and long-duration reliability situations that are similar to real-world use.

## Figures and Tables

**Table 1:** Dimensions of the four fingers in the prosthetic hand prototype.

Finger	Distal Phalanx (mm)	Middle Phalanx (mm)	Proximal Phalanx (mm)
Index finger	20	20	42
Middle finger	22	25	46
Ring finger	20	23	44
Little finger	20	20	32

**Table 2:** Dimensions of the thumb in the prosthetic hand prototype.

Thumb Linkage	Length (mm)
Distal phalanx	25
Proximal phalanx	30
Metacarpal bone	57

**Table 3:** Overall dimensions of the prosthetic hand prototype.

Hand Volume Parameters	Value (mm)
Width	71
Length	197
Thickness	33

**Table 4:** Specifications of the grasped objects.

Label	Object	Diameter/Size (mm)	Mass (g)
Object 1	Light bottle	55.62	22.13
Object 2	Medium bottle	55.62	200.27
Object 3	Heavy bottle	55.62	368.70
Object 4	Large sphere	70.40	32.32
Object 5	Large cylinder	40.54	8.37
Object 6	Small cylinder	28.67	27.81
Object 7	Plush toy	Irregular	42.58
Object 8	Small sphere	49.01	3.63
Object 9	Medium sphere	55.73	13.36
Object 10	Card	3.03 (thickness)	9.77

## Data Availability

The data used to support the findings of this study are available from the corresponding author upon reasonable request.
